# Salvage Pelvic Lymph Node Dissection in Recurrent Prostate Cancer: Surgical and Early Oncological Outcome

**DOI:** 10.1155/2015/198543

**Published:** 2015-01-28

**Authors:** Tom Claeys, Charles Van Praet, Nicolaas Lumen, Piet Ost, Valérie Fonteyne, Gert De Meerleer, Bieke Lambert, Louke Delrue, Pieter De Visschere, Geert Villeirs, Karel Decaestecker

**Affiliations:** ^1^Department of Urology, Ghent University Hospital, De Pintelaan 185, 9000 Ghent, Belgium; ^2^Department of Radiation Oncology and Experimental Cancer Research, Ghent University Hospital, 9000 Ghent, Belgium; ^3^Department of Nuclear Medicine, Ghent University Hospital, 9000 Ghent, Belgium; ^4^Department of Radiology, Ghent University Hospital, 9000 Ghent, Belgium

## Abstract

*Methodology*. Seventeen patients with prostate-specific antigen (PSA) rise following local treatment for prostate cancer with curative intent underwent open or minimally invasive salvage pelvic lymph node dissection (SLND) for oligometastatic disease (<4 synchronous metastases) or as staging prior to salvage radiotherapy. Biochemical recurrence after complete biochemical response (cBR) was defined as 2 consecutive PSA increases >0,2 ng/mL; and after incomplete biochemical response as 2 consecutive PSA rises. Newly found metastasis on imaging defined clinical progression (CP). Palliative androgen deprivation therapy (ADT) was initiated if >3 metastases were detected or if patients became symptomatic. Kaplan-Meier statistics were applied. *Results*. Clavien-Dindo grade 1, 2, 3a, and 3b complications were seen in 6, 1, 1, and 2 patients, respectively. Median follow-up time was 22 months. Among 13 patients treated for oligometastatic disease, 8 (67%) had a PSA decline, with 3 patients showing cBR. Median PSA progression-free survival (FS) was 4.1 months and median CP-FS 7 months. Three patients started ADT, resulting in a 2-year ADT-FS rate of 79.5%. *Conclusion*. SLND is feasible, but postoperative complication rate seems higher than that for primary LND. Biochemical and clinical response duration is limited, but as part of an oligometastatic treatment regime it can defer palliative ADT.

## 1. Introduction and Objectives

Primary treatments for localized prostate cancer (PC) are provided for optimistic oncological results, with even high-risk patients obtaining a 10-year cancer specific survival of 90% [1]. However, these outcomes are accompanied by a significant number of patients presenting with biochemical recurrence after primary treatment, indicating the presence of prostatic epithelial tissue. These 20–50% of cases that do progress with a rise in PSA might show reduced cancer-specific survival rates [2–4]. Advanced imaging techniques, such as magnetic resonance imaging (MRI) and choline positron emission tomography (PET) combined with computed tomography (CT), aim to detect recurrent disease in as early as possible stage [5].

Within this group large differences exist in clinical progression patterns and survival rates. Several groups recently demonstrated the prognostic importance of the site of recurrence, favoring nodal disease with a better outcome as compared to osseous or visceral spread [6, 7]. Furthermore, limited spread of disease, often referred to as oligometastatic disease, has also been associated with better oncological prognosis [6, 8, 9].

In the past nonlocalized PC was often initially treated systemically, mainly by means of androgen deprivation therapy (ADT). These treatments can cause an important decrease in quality of life, are not curative, and may eventually lead to castration resistant disease requiring further systemic treatment [10]. A nonsystemic treatment such as salvage lymph node dissection (SLND) might be able to halt further recurrence, whilst deferring systemic treatment [11]. Results from several SLND series show the possibility of a biochemical response after surgery, although its duration may vary [12–17]. On the downside, reported complication rates may have a significant impact on quality of life [16, 18].

In this light, we present our series of SLND, performed mostly with minimally invasive surgical techniques, in which we weigh the surgical morbidity against a potential oncological benefit, without the effect of adjuvant therapies. This paper further aims to describe the possibility of delaying systemic therapy within this patient group.

## 2. Materials and Methods

### 2.1. Patient Selection

Between June 2009 and June 2014, 17 patients underwent SLND at our institution. Indications for SLND were treatment of oligometastatic PC recurrence (<4 synchronous metastases) as visualized on standard CT with additional bone scan, fluorodeoxyglucose- (FDG-) PET-CT (*n* = 7), or choline PET-CT (*n* = 6) or staging prior to salvage prostate bed (pN0) or whole-pelvis (pN1) radiotherapy (*n* = 4). Median preoperative PSA was 2,01 ng/mL (range 0,24–26,5). The study was approved by the local ethics committee (EC UZG 2011/495).

### 2.2. Surgical Details

Surgery was performed open (*n* = 4), transperitoneally laparoscopic (*n* = 7), or robot-assisted (*n* = 6) by 2 surgeons (N.L. and K.D.C.). SLND template was bilaterally extended in 13 patients, including 4 patients with additional removal of suspicious lymph nodes (LNs) outside this template (presacral and para-aortic nodes), unilaterally extended in 1 patient, and limited to suspicious LNs on imaging in 3 patients. Extended SLND includes removal of all fatty, fibrous, and lymphatic tissue medially from the lateral border of the external iliac artery, laterally from the hypogastric artery, from the obturator fossa with complete deskeletonization of the obturator nerve, caudally up to and including the node of Cloquet, and cranially up to the crossing of the ureter over the common iliac artery. Titanium clips were used to seal distal lymphatic vessels before transection [19]. Finally, one drain was placed on each operated side in the obturator fossa and was usually removed when it produced <100 mL over 24 h. Standard thromboembolic prophylaxis comprised early mobilization, compression stockings, and 14 days of LMWH injected in the upper arm [19]. Sampled LNs from each specimen were fixed overnight in 4% buffered formalin, processed for paraffin embedding, and cut into 2 *μ*m thick sections. All sections underwent hematoxylin-eosin staining and microscopic evaluation by two urological pathologists.

### 2.3. Data Collection

Data on patient and tumor characteristics, surgery, and pathology were gathered prospectively. Complications were recorded during hospitalization and on follow-up visits (at least one within the first postoperative month). All patients had PSA measurement within 40 days following surgery and at intervals of 3 months thereafter. Imaging was performed at the treating physician's discretion. Postoperative complications were graded according to Clavien-Dindo classification [20]. A lymphocele was considered symptomatic when it caused local pain, fever, bladder neck obstruction, or thromboembolism or if the patient suffered lymphorrhea >5 days through the drain or percutaneous. Complete biochemical response (cBR) following SLND was defined as PSA < 0,2 ng/mL within 40 days postoperatively, whereas incomplete response (iBR) was defined as PSA > 0,2 ng/mL. Nonresponders did not show a drop in PSA. After cBR, biochemical recurrence was defined as an increase in PSA > 0,2 ng/mL on 2 consecutive measurements. After iBR, 2 consecutive PSA rises were considered as biochemical progression [13]. PSA progression was reached when either biochemical recurrence or progression was seen. At least one new metastatic site on postoperative imaging was defined as clinical progression (CP). Palliative androgen deprivation therapy (ADT) was initiated if >3 metastases were detected or if patients became symptomatic.

### 2.4. Statistical Analysis

All statistical analyses were performed using SPSS version 22.0 (IBM, Armonk, NY, USA). Values are presented as median (range). Kaplan-Meier statistics were applied to determine PSA progression-, CP- and ADT-free survival (FS). Kaplan-Meier graphs were produced using MEdCalc, version 14.10 (MEdCalc Software, Oostende, Belgium).

## 3. Results

Patient and tumor characteristics are described in further detail in Table 1. For the analysis of surgical outcomes all 17 patients were included. To evaluate the oncological outcome of SLND only patients that did not undergo SLND as part of a diagnostic work-up before salvage radiation therapy were regarded (*n* = 13).

### 3.1. Surgical Outcome (*n* = 17, Tables 2 and 3)

11 nodes as a median were resected (range 1–21), with 15 patients having histopathological confirmation of LN involvement. Median follow-up time was 22 months (range 4–61). Two laparoscopic interventions were converted to open surgery because of extensive adhesions. Grade 1, 2, 3a, and 3b complications were seen in 6 (33%), 1 (6%), 1 (6%), and 2 (11%) patients, respectively. Three patients suffered transient penile or scrotal edema, 2 had a temporary loss of sensation in the genitofemoral nerve dermatome, and 1 showed prolonged ileus after surgery (grade 1). Of those requiring further treatment, one patient was admitted for antibiotic treatment because of a hospital-acquired pneumonia (grade 2) and 3 patients underwent additional interventions to cope with surgical complications: one patient had a percutaneous drainage of a lymphocele and received anticoagulants for a deep venous thrombosis (grade 3a); another presented with partial bladder necrosis demanding transurethral resection; finally a lymphocele with abscedation needed to be surgically drained under general anesthesia (grade 3b). All of grade 3a or 3b complications were reported after open SLND.

### 3.2. Oncological Outcome (*n* = 13)

**Figure 1:**
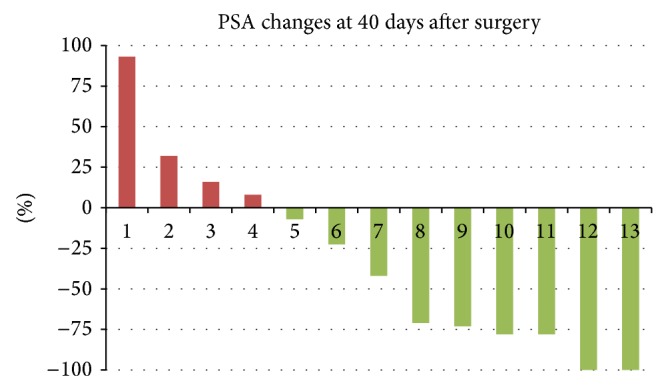
Percentual change in PSA within 40 days postoperatively, in patients treated for oligometastatic disease.

**Figure 2:**
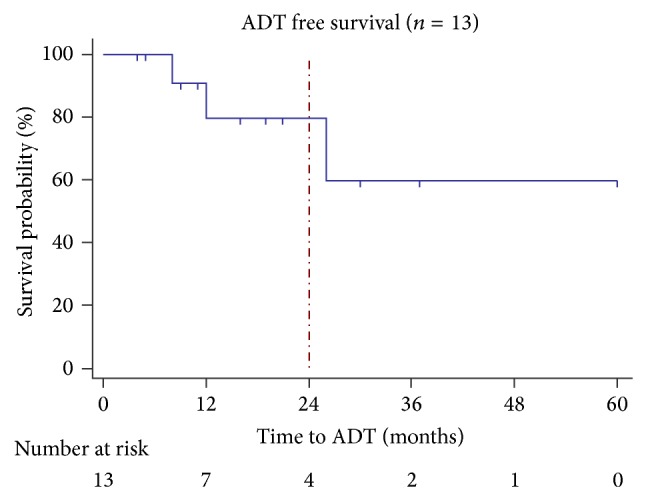
Post-SLND ADT-free survival among patients treated for oligometastatic disease (*n* = 13). Projected 2-year ADT-FS is 79.5%.

Among the 13 patients treated for oligometastatic disease, all but one with histopathological LN involvement, 9 (69%) had a postoperative PSA decline, including 3 patients (23%) that showed cBR (Figure 1). Median follow-up was 21 months (range 4–60) in the oncological group. Median PSA progression-FS was 4.1 months (range 0–20). Eight patients (54%) showed CP: 1 solely to pelvic LNs outside SLND template, 1 to pelvic LNs outside SLND template and to the bone, 3 to retroperitoneal LNs, 1 to the bone, 1 to the penile spongious body, and 1 to the abdominal wall. Median CP-FS was 7 months (range 3–38). Of patients without clinical progression, 3 still have cBR and 2 showed biochemical recurrence. Five (38%) and 1 (8%) patients were treated with 1 and 2 additional oligometastasis-directed therapies, respectively. These include surgical resection of a metastasis in the penile spongious body and of an abdominal wall mass, stereotactic body radiation therapy (SBRT) to a vertebral lesion, SBRT for a bone lesion with a synchronous positive pelvic LN, and three times SBRT for positive LNs (both pelvic and para-aortic). Three patients (23%) started palliative ADT, resulting in a projected 2-year ADT-FS rate of 79.5% (Figure 2).

## 4. Discussion

### 4.1. Surgical Outcome

The surgical complication rate of this series of SLND is far from trivial, with 59% of patients reporting adverse events, even though most of them are grade 1 as defined by the Clavien-Dindo classification. This is in accordance with other series of SLND reporting overall complication rates ranging from 0 to 90% [13, 15, 16, 21]. The most frequently reported complications in literature are lymphorrhea, fever, and prolonged ileus. The only reported grade 3b complication was a lymphocele requiring surgical drainage, as described by Suardi et al. [16] We can thus conclude that both the pattern and the frequency of adverse events we report here are consistent with other series. Noteworthy is the fact that this is the first prospective series to date reporting surgical and oncological outcomes of SLND performed mostly with minimal invasive techniques. All of our grade 3a and 3b complications were seen after open SLND, hinting that the use of minimally invasive techniques might reduce surgical morbidity in this setting, including wound dehiscence and prolonged ileus. More, larger and comparative series are needed to determine the optimal SLND approach.

Notably, complications seem to be more frequent in the salvage setting than in the primary treatment setting. We recently published data on surgical complications of PLND performed by the same surgeons prior to radiotherapy: primary staging PLND had an overall complication rate of 25%, with 11%, 2%, 4%, and 5% grade 1, 2, 3a, and 3b events [22]. The 2- to 3-fold higher complication rate following SLND might be attributed to previous pelvic treatment (both surgery and/or radiation therapy) causing local fibrosis and blurring surgical landmarks.

### 4.2. Oncological Outcome

In our series, median time to biochemical recurrence (after complete PSA response) or progression (after incomplete PSA response) was only 4.1 months. Of other published series, only the group of complete responders of Jilg et al. can be compared to this group since these patients also did not receive adjuvant ADT and since the same biochemical cutoffs were used. They report a median time to biochemical progression and recurrence of 9,8 and 16,7 months, respectively, but it is unclear whether this group received adjuvant radiation therapy or not [13]. We report a median clinical progression-free survival of 7 months without adjuvant therapy, whereas other series report 15 to 60 months until clinical progression, but with adjuvant therapy [13, 16, 21]. We succeeded in our aim of deferring systemic treatment, as ADT was only initiated in 3 patients. The projected ADT-FS at 2 years is 78.5%, which exceeds other oligometastatic treatment regimes with stereotactic body radiation therapy, showing a 2-year ADT-FS of 50% [23]. Deferring systemic treatment might improve overall quality of life, but the final oncological effects are yet to be shown. However, several trials are being conducted to clarify these outcomes (NCT01558427, NCT01777802, and NCT01859221).

Our cohort represents a diverse group of patients with heterogeneous primary histology (Gleason 6–9) and PSA levels at the time of SLND ranging from 0,69 to 26,54 ng/mL. On this matter, patients with lower PSA and a Gleason score below 8 at radical prostatectomy might have a better outcome than others after SLND [13, 16, 24]. Better patient selection might improve oncological outcome of SLND and would only expose those patients that might have a substantial benefit to the possible surgical morbidity.

## 5. Conclusions

This series presents surgical and early oncological results after SLND without the effect of any adjuvant therapies, be it ADT or radiation therapy. SLND for oligometastatic PC recurrence seems feasible, both open and minimally invasive, but postoperative complication rate is rather high as compared to primary PLND series. Although only a limited number of patients had a durable biochemical response, as part of an oligometastatic treatment regime it can defer palliative ADT. Larger, prospective trials are needed for further clarification of these results.

## Figures and Tables

**Table 1:** Patient and tumor characteristics for all patients and patients in oncological follow-up.

	All patients (surgical follow-up) (*n* = 17)	Oncological follow-up (*n* = 13)
Median age, years (range)	65 (48–77)	65 (48–77)
Median body mass index, kg/m^2^ (range)	28,9 (23,3–32,7)	24,7 (23,3–32,7)
Median PSA at diagnosis, ng/mL (range)	12 (5,2–38)	12 (5,9–38)
Gleason score, *n* (%)		
6	1 (6)	1 (8)
7 (3 + 4)	5 (29)	4 (31)
7 (4 + 3)	3 (18)	2 (15)
8	5 (29)	4 (31)
9	3 (18)	2 (15)
10	—	—
Clinical tumor stage, *n* (%)		
T2	6 (35)	5 (38)
T3a	4 (24)	4 (31)
T3b	3 (18)	1 (8)
T4	1 (6)	1 (8)
Unknown	3 (18)	2 (15)
Primary treatment, *n* (%)		
Surgery	14 (82)	11 (85)
Radiotherapy	2 (12)	2 (15)
High-intensity, focused ultrasound	1 (6)	—
Previous PLND, *n* (%)	5 (29)	—
Median PSA at SLND, ng/mL (range)	2,01 (0,24–26,54)	2,01 (0,69–26,54)
Median PSAdt at SLND, months (range)	5,3 (1,5–46,5)	5,1 (1,6–19,8)
Median follow up, months (range)	22 (4–60)	21 (4–60)

^*^PSA = prostate specific antigen; PSAdt = PSA doubling time; PLND = pelvic lymph node dissection; SLND = salvage pelvic lymph node dissection.

**Table 2:** Surgical details (*n* = 17).

Surgical approach	4 (24%)	Open
	7 (41%)	Laparoscopic
	6 (35%)	Robot-assisted

Resected nodes		
Total number of resected nodes	200	
Total number of positive nodes	39	
Number of resected nodes/patient (median, range)	11 (1–21)	
Number of positive nodes/patient (median, range)	1 (0–6)	

Distribution of nodes (%)	**Resected (*n*** = **200)**	**Positive (*n*** = **39)**
Common iliac nodes	5%	5%
Internal iliac nodes	19%	23%
External iliac nodes	42%	38%
Obturator nodes	31%	18%
Presacral nodes	3%	3%
Para-aortic nodes	1%	3%
Pararectal nodes	2%	10%

SLND indication	13 (76%)	Treatment of oligometastatic disease
	4 (24%)	Staging prior to salvage radiotherapy

**Table 3:** Surgical complications according to Clavien-Dindo classification (*n* = 17).

None	7 (41%)	—	

Grade 1	6 (35%)	Transient penile/scrotal lymphedema: no treatment	3
		Sensitivity loss, inner thigh (genitofemoral nerve)	2
		Prolonged ileus	1

Grade 2	1 (6%)	Pneumonia (antibiotics)	1

Grade 3a	1 (6%)	Deep venous thrombosis due to lymphocele: percutaneous drainage + anticoagulants	1

Grade 3b	2 (12%)	Partial bladder necrosis: transurethral resection	1
		Abcedated lymphocele: surgical fenestration and drainage	1
